# Mitochondrial deficiency: a double-edged sword for aging and neurodegeneration

**DOI:** 10.3389/fgene.2012.00244

**Published:** 2012-11-26

**Authors:** Kostoula Troulinaki, Daniele Bano

**Affiliations:** German Center for Neurodegenerative DiseasesBonn, Germany

**Keywords:** aging, insulin/IGF-1, mitochondria, neurodegeneration, oxidative stress, TOR

## Abstract

For decades, aging was considered the inevitable result of the accumulation of damaged macromolecules due to environmental factors and intrinsic processes. Our current knowledge clearly supports that aging is a complex biological process influenced by multiple evolutionary conserved molecular pathways. With the advanced age, loss of cellular homeostasis severely affects the structure and function of various tissues, especially those highly sensitive to stressful conditions like the central nervous system. In this regard, the age-related regression of neural circuits and the consequent poor neuronal plasticity have been associated with metabolic dysfunctions, in which the decline of mitochondrial activity significantly contributes. Interestingly, while mitochondrial lesions promote the onset of degenerative disorders, mild mitochondrial manipulations delay some of the age-related phenotypes and, more importantly, increase the lifespan of organisms ranging from invertebrates to mammals. Here, we survey the insulin/IGF-1 and the TOR signaling pathways and review how these two important longevity determinants regulate mitochondrial activity. Furthermore, we discuss the contribution of slight mitochondrial dysfunction in the engagement of pro-longevity processes and the opposite role of strong mitochondrial dysfunction in neurodegeneration.

## INTRODUCTION

Eukaryotic cells have adopted an elaborated set of molecular mechanisms that prevent the accumulation of aberrant macromolecules (Kirkwood, 2005; Douglas and Dillin, 2010; Kourtis and Tavernarakis, 2011). Over time, these protective responses decline and make cells more vulnerable to stressful conditions. The consequent dysfunction of tissues and organs can prompt to the development of pathologies that compromise survival. For many years, the age-related decline was considered simply a passive and inevitable process. Conversely, it is now clear that aging is a biological process, which like many others is subjected to the regulation of well-defined signaling pathways (Kenyon, 2010; Bano et al., 2011; Martin, 2011). Most of these molecular cascades control metabolism, proliferation, stress resistance, and cell maintenance. Although their contribution to longevity was firstly described in simple model organisms with a relatively short lifespan, like yeast and invertebrates, a large number of findings in mammals support that they are evolutionary conserved and likely relevant in humans (Fontana et al., 2010).

Aging has a significant impact in our modern human society, as it is associated with the increased susceptibility to pathologies. Intensive studies in the last years have shown that most of the mechanisms involved in longevity influence also the onset of sporadic forms of brain disorders (Mattson, 2006; Mattson and Magnus, 2006; Bishop et al., 2010). The complex network of interactions intimately links various signaling pathways and molecular players that, together, contribute to such neurological conditions. Among them, mitochondria have a fundamental role in neuronal function and decline in their activity accelerates the onset and progression of age-related dysfunction (Nunnari and Suomalainen, 2012; Rugarli and Langer, 2012). Interestingly, while mild mitochondrial impairment extends the lifespan in various organisms as different as yeast, invertebrates and mice, significant suppression of mitochondrial activity compromises animal survival. Similarly, whilst mitochondrial deficiency or uncoupling can partially delay neuronal degeneration as a result of excitotoxic injury or toxins, loss-of-function mutations in genes encoding certain mitochondrial proteins can negatively disturb neural circuits and ultimately lead to cell death. Here, we review the advances in understanding some of the molecular mechanisms that regulate certain aspects of aging, such as age-related mortality. We also dedicate particular attention to the contribution of mitochondria to the signaling pathways involved in this important biological process. Moreover, we address the controversial opposite role of mitochondrial dysfunction in the onset of brain pathologies.

## LONGEVITY PATHWAYS

### THE INSULIN/IGF-1 SIGNALING PATHWAY

**FIGURE 1:**
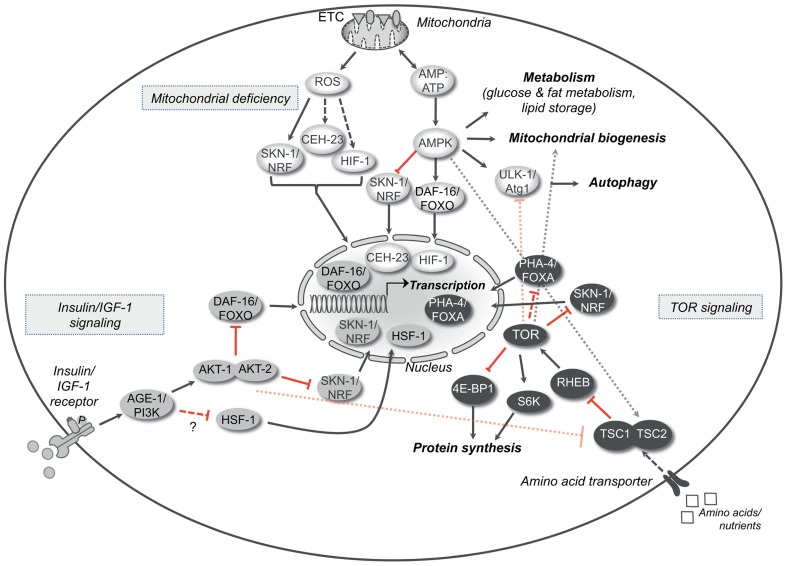
**Longevity pathways**. The insulin/IGF-1 signaling, the TOR signaling and the molecular cascade that is activated by mild mitochondrial dysfunction are three important pathways that interact with each other and modulate aging in various organisms. The crosstalk between these pathways is represented in the figure. Arrows indicate positive regulatory events and red bars indicate inhibitory interactions. Dotted arrows or bars represent interactions between the different pathways, whereas dashed arrows or double bars indicate possible indirect interactions. AGE-1/PI3K, phospatidylinositol-3-kinase; DAF-16/FOXO, forkhead box O (FOXO) transcription factor; HSF-1, heat shock response transcription factor-1; SKN-1/Nrf, skin in excess transcription factor 1/NF-E2-related factor; TSC1/2, tuberous sclerosis complexes 1 and 2; RHEB, Ras homolog enriched in brain; TOR, target of rapamycin kinase; S6K, S6 kinase; 4E-BP1, eukaryotic initiation factor 4E-binding protein; PHA-4/FOXA, forkhead box A (FOXA) transcription factor; ROS, reactive oxygen species; ATP, adenosine-5′-triphosphate; CEH-23, homeobox transcription factor; HIF-1, hypoxia-inducible transcription factor 1; AMPK, adenosine monophosphate-activated protein kinase; ULK-1/Atg1, serine-threonine kinase ortholog of the autophagy related kinase 1 (Atg1).

The insulin/IGF-1 signaling pathway is one of the main pathways regulating aging in organisms ranging from invertebrates, like *Drosophila melanogaster* and *Caenorhabditis elegans*, to mammals. The role of this pathway in longevity was initially identified in the nematode *C. elegans* through the discovery of mutants that decrease the activity of the pathway and extend the lifespan of the organism (Kenyon et al., 1993). The existence of such mutations supported the concept of molecular factors underlying aging. Among them, mutations in the gene *age-1* extend the chronological lifespan of the nematode (Friedman and Johnson, 1988). This gene encodes the *C. elegans* ortholog of the class I phosphoinositide 3-kinase (PI3K) and is a key enzyme in the Insulin/IGF-1 signaling pathway. It catalyzes the production of phosphatidylinositol-3,4,5-trisphosphate (Morris et al., 1996) that serves as a second messenger for the activation of downstream kinases. AGE-1/PI3K is activated by the sole insulin/IGF-1 receptor DAF-2, which belongs to the tyrosine kinase receptor family and is a master regulator of metabolism. Mutations in the *daf-2* gene almost double the lifespan of nematodes (Kenyon et al., 1993), mainly through the activation of the transcription factors DAF-16/FOXO, SKN-1/Nrf, and HSF-1 (Hsu et al., 2003; Tullet et al., 2008; **Figure 1**). In animals with reduced insulin/IGF-1 signaling, the nuclear translocation of DAF-16/FOXO, SKN-1/Nrf, and HSF-1 promotes the expression of various target genes involved in stress resistance, proteostasis, defense reaction and metabolism (Narasimhan et al., 2009). Interestingly, enhanced transcription in certain tissues contributes differently to the aging of somatic tissues. For example, specific expression of *daf-16* in the intestine – the main adipose tissue in nematodes – extends the lifespan of *daf-16*; *daf-2* double mutants, although it is not sufficient to completely restore the same survival as in the *daf-2* mutant animals (Libina et al., 2003). Notably, the activity in one tissue, like in the case of the intestine, can regulate DAF-16-mediated longevity pathways in others in a feedback loop that controls post-mitotic cell senescence (Murphy et al., 2007). In this context, the intestinal DAF-16/FOXO coordinates the rate of aging of the whole organism in response to signals from the reproductive and nervous systems. Block of germ cell proliferation in animals lacking functional gonad increases the lifespan through the DAF-16/FOXO accumulation in the intestinal nuclei and the consequent gene transcription (Lin et al., 2001; Arantes-Oliveira et al., 2002). Remarkably, loss-of-function of the microRNA *mir-71* in the nervous system suppresses intestinal DAF-16-dependent gene expression and therefore germline-mediated longevity (Boulias and Horvitz, 2012), further underlying the complexity of the signals that dictate how long an organism is going to live.

The prominent role of the insulin/IGF-1 signaling pathway in longevity is evolutionary conserved across species. In *D. melanogaster*, mutations in the sole insulin/IGF-1 receptor (dINS) or the insulin receptor substrate *chico* extend the lifespan through the activation of the FOXO transcription factor (Clancy et al., 2001; Tatar et al., 2001; Slack et al., 2011). Similarly to nematodes, FOXO overexpression in the fat body is sufficient to increase the lifespan of flies (Giannakou et al., 2004; Hwangbo et al., 2004). In mice, haploinsufficiency of the insulin-like growth factor type 1 receptor (Igf1r) significantly increases the lifespan compared with wild-type littermates (Holzenberger et al., 2003). Although the recent findings argue the increased longevity of Igf1r-deficient mice (Bokov et al., 2011), it is accepted that mild reduction of the insulin/IGF-1 signaling throughout the body or even restricted at the central nervous system can increase the lifespan of mice (Taguchi et al., 2007). Even in humans, accumulating evidence suggests that lower insulin/IGF-1 signaling is beneficial for longer survival (van Heemst et al., 2005). It is noteworthy to mention that single nucleotide polymorphisms in FOXO3A gene are strongly associated with human longevity (Willcox et al., 2008). Likewise, a study on centenarians demonstrated that heterozygous mutations in the highly polymorphic *Igf1r* are correlated with longevity in humans (Suh et al., 2008).

Decreased activity of the insulin/IGF-1 signaling pathway enhances the resistance to exogenous and endogenous oxidative stress in nematodes as well as in mice (Holzenberger et al., 2003; Hsu et al., 2003). This is the result of the DAF-16/FOXO reprogramming process and the consequent synthesis of chaperones and other anti-oxidant factors. Recent evidence suggests that mitochondria are also important for the resistance of *daf-2* mutant nematodes through the production of reactive oxygen species (ROS). ROS are a by-product of oxidative phosphorylation but apart from their toxic effect in high concentrations, they can also act as signaling molecules. At least in nematodes, impairment of the insulin/IGF-1 pathway increases mitochondrial activity and, as a consequence, ROS production. ROS mediate a retrograde response resulting to up-regulation of genes encoding antioxidant enzymes. Importantly, AMP-activated protein kinase (AMPK) is required to sense the intracellular energetic status associated with enhanced oxidative stress. As a result, AMPK up-regulates L-proline mitochondrial catabolism while reduces glucose metabolism, further contributing to the ROS generation (Ristow and Zarse, 2010; Zarse et al., 2012). In parallel, while AMPK controls mitochondrial respiration in impaired glucose conditions, SKN-1(Nrf) and PMK-1(p38) up-regulate the transcription of specific genes and induce the protective stress resistance response. Clearly, AMPK is a fundamental intracellular checkpoint as it adapts intracellular metabolism and catabolism to the energetic needs of the organism. In *C. elegans*, AMPK is required for the extension of lifespan in mutants with reduced insulin/ IGF-1 signaling, as it controls the lipid storage and the fat metabolism according to the energetic stress (Apfeld et al., 2004; Curtis et al., 2006; Narbonne and Roy, 2009). Overexpression of the AMP-activated protein kinase subunit AAK-2 prolongs the lifespan of the organism, whereas its loss-of-function reduces it (Apfeld et al., 2004). Notably, AMPK deficiency compromises the rapid mobilization of fat reservoirs, leading to premature lethality of dauer larvae. Recently, it was revealed that AMPK acts on catalases and regulates the levels of H_2_O_2_ into the cell (Xie and Roy, 2012). Similarly to ROS, sub-lethal doses of H_2_O_2_ signal to the nucleus through the HIF-1 transcription factor and modulate the physiology of the cell enabling the survival under stress. Some of the genes that are up-regulated favor the biosynthesis of fatty acids. In parallel, H_2_O_2_ blocks lipases and protects lipid stores. Moreover, AMPK modulates autophagy through the direct phosphorylation of ULK1, the mammalian ortholog of Atg1, as a required step for survival when nutrients are insufficient (see next paragraph and Lee et al., 2010a; Egan et al., 2011; Kim et al., 2011).

Although it is currently unknown whether other mitochondria-to-nucleus signals are engaged in insulin/IGF-1 deficient organisms, the regulation of mitochondrial respiration is clearly a key component of lifespan extension and can further influence survival. As an example, increased mitochondrial fusion, by suppressing dynamin-related protein DRP-1 expression does not alter the lifespan of wild-type animals, whereas it further prolongs the survival of *daf-2* mutant nematodes (Yang et al., 2011). Similarly, increased mitochondrial proliferation in insulin/IGF-1 deficient animals as a result of some genetic lesions, like prohibitins, causes a twist in cellular metabolism and further extends the lifespan of nematodes (Artal-Sanz and Tavernarakis, 2009).

### THE TOR SIGNALING PATHWAY

The serine/threonine kinase “target of rapamycin” TOR (mTOR in mammals) has drawn large attention for its pleiotropic effects on aging through the control of multiple downstream pathways (Ravikumar et al., 2010; **Figure 1**). TOR senses the availability of amino acids and nutrients into the cell and regulates cell growth, proliferation, and metabolism accordingly. In the presence of growth factors, like insulin and IGF-1, Akt kinase is activated and controls the function of the TSC1/TSC2 complex, a negative regulator of mTOR. Post developmental TOR inhibition extends the lifespan of many different organisms, ranging from yeast to mammals (Vellai et al., 2003; Jia et al., 2004; Kapahi et al., 2004; Kaeberlein et al., 2005; Harrison et al., 2009). Moreover, TOR mediates gene transcription that, at least in yeast and in *C. elegans*, is necessary for the effect on chronological lifespan (Medvedik et al., 2007; Sheaffer et al., 2008). In yeast, TOR increases the expression of the nicotinamidase gene *PNC1*, an important regulator of the NAD-dependent deacetylase Sir2, through the transcription factors Msn2p and Msn4p (Medvedik et al., 2007). In *C. elegans*, reduced TOR signaling enables the forkhead transcription factor PHA-4 to induce the expression of pro-survival factors that contribute to lifespan extension of animals under nutrient restriction (Sheaffer et al., 2008). In a variety of conditions, TOR signaling controls the expression of stress-resistance genes through the SKN-1/Nrf transcription factor and prevents the ROS formation beyond a fatal threshold due to the increased mitochondrial metabolism (Robida-Stubbs et al., 2012; Zarse et al., 2012). In mammals, decreased mTOR activity, due to rapamycin, prevents the direct binding and coactivation of the transcription factor ying-yang 1 (YY1) with the peroxisome proliferator-activated receptor gamma coactivator 1-alpha (PGC-1α). At the molecular level, disruption of this complex and reduced recruitment to the promoters of genes encoding mitochondrial proteins diminishes mitochondrial biogenesis and consequently oxidative phosphorylation (Cunningham et al., 2007).

Beside the regulation of gene expression, TOR pathway contributes to aging through its role in protein synthesis: TOR activates the ribosomal subunit S6 kinase (RS6K) and in parallel inhibits the 4E-BP1, which is a negative regulator of translation, resulting in increased protein synthesis. Block of protein synthesis through inhibition of RS6K or the initiation of translation 4E protein (eIF4E), which is the target of 4E-BP inhibitor, leads to lifespan extension in various organisms (Kapahi et al., 2004; Kaeberlein et al., 2005; Hansen et al., 2007; Pan et al., 2007; Syntichaki et al., 2007; Selman et al., 2009). In S6K knockout mice they have found activation of pathways regulated by PGC-1α and AMPK in some tissues, like the liver, adipose tissue, or muscles (Selman et al., 2009). These pathways modulate mitochondrial biogenesis. In yeast, Sch9/S6K regulates mitochondrial oxygen consumption and mutant strains, either for TOR or Sch9/S6K, up-regulate both nuclear and mitochondrial genes encoding proteins of oxidative phosphorylation (OXPHOS; Pan and Shadel, 2009). In flies kept under dietary restriction (DR), the 4E-BP boosts mitochondrial activity due to enhanced translation of nuclear-encoded mitochondrial genes, whereas inhibition of the electron transport chain prevents the lifespan extension (Zid et al., 2009).

By all means, the integration of the cellular status depends on the crosstalk between the different pathways and intracellular sensors. Nutrient or growth factor deprivation promotes the catabolic process autophagy through TOR. Autophagy is a homeostatic process likely developed in unicellular organisms as an adaptive survival response to harsh conditions (Yorimitsu and Klionsky, 2005; Singh and Cuervo, 2011). It is important for the turnover of intracellular macromolecules and damaged organelles and, it is widely considered as a potential anti-aging mechanism. Thus, in the case of mitochondria, TOR not only regulates mitochondrial biogenesis but also regulates mitochondrial turnover though macroautophagy (mitophagy). During this process the cytosolic material is engulfed by double-membrane vesicles and targeted to the lysosome for degradation. This quality control mechanism protects from the intracellular accumulation of dysfunctional organelles and, therefore, from eventual oxidative stress as a result of inefficient oxidative phosphorylation. TOR modulates autophagy through a cascade of events that alters the phosphorylation status of the serine/threonine kinases ULK-1/ULK-2, the mammalian orthologs of Atg1, and the association between Ambra1 and Beclin-1, favoring the recruitment of autophagy-related proteins to the nascent phagophore (Rubinsztein et al., 2011). Notably, reduction of TOR activity increases autophagy, which is required for the lifespan extension in TOR-deficient animals and insulin/IGF-1 defective mutants (Vellai et al., 2003; Hansen et al., 2008; Toth et al., 2008; Bjedov et al., 2010). According to this view, block of autophagy abolishes the extension of lifespan in the *daf-2* mutants independently of the DAF-16/FOXO transcription factor, although with a less pronounced effect compared to *daf-16* loss-of-function (Melendez et al., 2003; Hansen et al., 2008). Possibly, enhanced autophagy promotes longevity only in those conditions in which the engagement of the nuclear expression machinery directs raw material deriving from catabolic processes to newly synthesized biomolecules. The role of TOR pathway in aging is further supported by studies showing that the TOR inhibitor rapamycin prolongs the lifespan of different organisms through changes in the protein synthesis and autophagy (Kapahi et al., 2004; Kaeberlein et al., 2005; Hansen et al., 2008; Toth et al., 2008; Harrison et al., 2009; Bjedov et al., 2010). Interestingly, rapamycin treatment protects from some age-related pathologies, such as cancer, and extends the lifespan of mice, even when the feeding begins during adulthood. This might lead to the development of pharmacological interventions targeting mTOR signaling, which could theoretically delay some of the age-related phenotypes and prevent age-related disorders (Harrison et al., 2009).

### MITOCHONDRIAL DEFICIENCY AND OXIDATIVE STRESS

One of the first and most accepted aging theories, called the “free radical theory of aging,” proposes that loss of protective mechanisms and enhanced ROS-dependent macromolecule’s damage create a vicious cycle that leads to progressive deterioration of the intracellular systems (Harman, 1956). At the cellular level, insufficient handling of oxidative stress induces senescence and ultimately death. According to this theory, mitochondria contribute as the main source of intracellular ROS, which then cause age-related decline of respiration through damage of the ETC subunits (Kirkwood, 2005). The gradual leakage of the mitochondrial electron transport system is the main endogenous source of reactive radicals that sustains this deleterious feedback loop. In addition, as a consequence of uncontrolled oxidative stress, mitochondrial DNA (mtDNA) accumulates many mutations or deletions. Interestingly, studies in cell lines have revealed that mitochondria with impaired ETC or mtDNA mutations can produce even more ROS further increasing the ROS overload of the cell (Indo et al., 2007). Other studies have shown that mutations in the mtDNA accumulate during aging and, at least in mice, can accelerate certain age-related phenotypes (Melov et al., 1995a,b; Welle et al., 2003). However, whether mtDNA mutations are the cause or the consequence of aging is still a matter of debate. In a mouse model expressing an error-prone version of the catalytic subunit of the mtDNA polymerase, accumulation of mtDNA mutations leads to respiratory dysfunction and premature aging (Trifunovic et al., 2004). Interestingly, these animals do not show increased ROS production indicating that their accelerated aging might be linked to respiratory deficiency rather than oxidative stress (Trifunovic et al., 2005). In line with these observations, the use of vitamins, natural antioxidants, does not have any effect in the life expectancy in humans (Bjelakovic et al., 2008; Chong-Han, 2010). Even more intriguing is the fact that nematode mutants for the superoxide dismutase (SOD) genes show prolonged rather than decreased lifespan, despite the significant oxidative damage (Van Raamsdonk and Hekimi, 2009). All these indications raise questions whether oxidative stress is the main cause of aging or it is simply the result of extended mitochondrial dysfunction (Hekimi et al., 2011).

## MITOCHONDRIAL FUNCTION AND AGING: HOW TO LIVE LONGER

**FIGURE 2:**
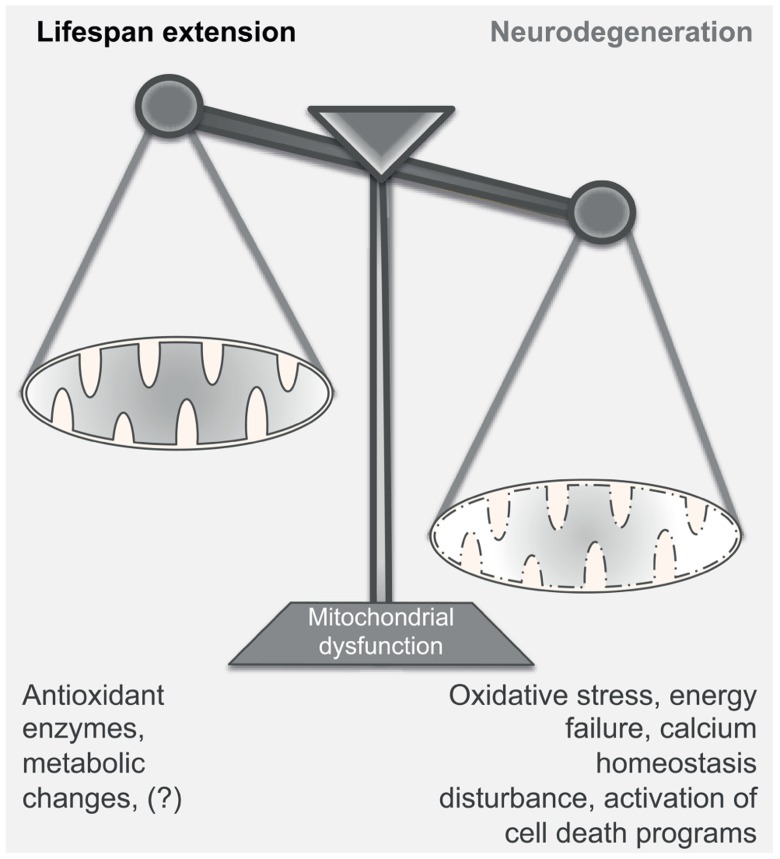
**Mitochondrial dysfunction in aging and neurodegeneration**. Mitochondrial dysfunction can have either a positive or a negative effect on the normal function of the cell; mild mitochondrial dysfunction stimulates retrograde signaling, increasing – among others – the production of antioxidant enzymes and inducing metabolic changes. This adaptive response leads to the extension of the lifespan of the organism. On the contrary, severe disturbance of the mitochondrial activity may become detrimental for the survival of the cell, as it compromises energy production, induces oxidative stress and disturbs calcium homeostasis. Under these conditions, neurons undergo rapid changes of the dendritic structures and, according to the damage, can engage detrimental programs that cause cell death. Thus, strong mitochondrial inefficiency may lead to the development of neurodegenerative diseases.

Efficient oxidative phosphorylation is critical for the normal cellular function as it provides most of the intracellular energy. Paradoxically, slight mitochondrial dysfunction exerts a beneficial effect on the lifespan in many organisms. Indeed, RNA interference (RNAi) or mutations in genes encoding certain subunits of the electron transport chain (ETC) cause mild mitochondrial defect and promote longevity. One example is the *clk-1* gene encoding a mitochondrial hydroxylase necessary for the ubiquinone biosynthesis and therefore important for an effective ETC. Both in *C. elegans* and in mice, mutation or haploinsufficiency of *clk-1* decreases the oxidative phosphorylation rate and prolongs significantly the lifespan (Lakowski and Hekimi, 1996; Felkai et al., 1999; Liu et al., 2005). In nematodes, mitochondrial deficiency is associated with a delayed developmental rate, reduced adult size, and lower fecundity. The lifespan extension requires AMPK activity and the engagement of autophagy, whereas it is independent of the insulin/IGF-1 signaling pathway (Curtis et al., 2006; Toth et al., 2008; **Figure 1**). Longevity is also increased by altering mitochondrial function through silencing of genes encoding other mitochondrial proteins beside the ETC, as long as the treatments occur during development (Felkai et al., 1999; Dillin et al., 2002; Lee et al., 2003). However, null mutations in genes encoding ETC components severely compromise survival. Taken together, mitochondrial dysfunction can improve the fitness and survival of an organism up to a certain threshold beyond which toxicity is reached and viability is compromised (**Figure 2**). Even when restricted to a single tissue, like the intestine or the nervous system, mitochondrial dysfunction can extend the lifespan of the whole organism (Durieux et al., 2011). According to this model, mitochondrial stress in a limited number of cells is sufficiently sensed by surrounding tissues and modulates aging in a cell-non-autonomous manner. Although the pro-longevity signals remain to be identified, it is not excluded that ROS take part in the process, as antioxidants can limit this phenotype. Mitochondrial deficiency can engage protective pathways through gene transcription. In the case of long-lived animals, increased levels of ROS, along with decreased mitochondrial respiration, is sufficient to activate transcription factors, such as SKN-1 (An and Blackwell, 2003), CEH-23 (Walter et al., 2011), and HIF-1 (Lee et al., 2010b), that mediate the transcription of antioxidant enzymes like SOD, catalase, and glutathione transferase (**Figure 1**). These detoxifying enzymes can maintain the ROS levels below a certain threshold, protecting the cellular structures from extensive damage. This type of retrograde signaling is called mitochondrial hormesis (Ristow and Zarse, 2010) and is in accordance with the basic concept that the exposure of an organism to mild stress results in an adaptive or hormetic response (Calabrese and Baldwin, 2002). Beside the increased resistance to stress, another possible scenario might include the engagement of alternative metabolic pathways that sustain cellular functions (Liu and Butow, 2006). In support of this hypothesis, it has been found that activated AMPK induces the phosphorylation of DAF-16/FOXO and CHR-1/CREB in nematodes, mediating the expression of genes involved in metabolism and energy homeostasis (Greer et al., 2007b; Mair et al., 2011). In conclusion, similarly to other pro-longevity signaling pathways, mitochondrial deficiency could stimulate gene expression and change the consequent transcriptional profiles in response to altered ETC efficiency.

## MITOCHONDRIAL FUNCTION AND NEURODEGENERATION: A DELICATE BALANCE THAT CAN KILL

Mitochondria take part in a variety of heterogeneous intracellular processes. Specifically, they provide most of the cellular ATP through oxidative phosphorylation, produce ROS as side products, contribute to intracellular calcium homeostasis and under certain conditions, they can activate specific cell death programs. In neuronal cells, the abundance of mitochondria in subdomains critically regulates the density of dendritic structures, contributing to synaptic plasticity. Impairment of mitochondrial dynamics at the dendrites negatively affects the formation of new spines and leads to loss of synapses (Li et al., 2004). As it is expected, decline of the mitochondrial activity over time can progressively perturb the intracellular environment and affect the maintenance of the surrounding tissues. Thus, it is not surprising that aging and neurodegeneration are strongly linked with mitochondrial defects. However, at which rate mitochondrial activity enables survival and, conversely, at which degree compromised organelles cause irreversible damage remain two fascinating open questions. This possible double-edged sword aspect is of particular interest as mitochondria have apparently an opposite role in these two biological processes: while their severe dysfunction provokes neurodegeneration, a slight decrease in respiration extends the lifespan in a range of organisms as diverse as yeast, invertebrates, and mammals (**Figure 2**). Whether the engagement of pro-survival programs, including those activated by slight mitochondrial deficiency, can have any protective effect in brain disorders remains still unclear.

As previously shown in animals models, the use of inhibitors of the mitochondrial respiratory complexes induces neuronal degeneration in certain brain regions and therefore resembles certain types of pathologies. For example, the use of the neurotoxins rotenone and MPTP, which mainly act at the level of the Complex I, triggers the loss of dopaminergic neurons and causes symptoms similar to the sporadic forms of Parkinson’s disease (Gerlach et al., 1991; Panov et al., 2005). Similarly, the succinate dehydrogenase inhibitor 3-nitropropionic acid triggers extensive neurodegeneration in the striatum and has been used to model Huntington’s disease (Brouillet et al., 1999). In support of the mitochondrial role in brain disorders, a large number of studies have demonstrated a significant association between familial forms of neurodegenerative diseases and rare mutations in genes encoding proteins related to mitochondria. Interestingly, almost one third of the mutations that are linked to brain pathologies affect proteins required for the normal mitochondrial functions (Schon and Przedborski, 2011; Exner et al., 2012). Although Alzheimer’s, Parkinson’s and other neurodegenerative diseases are frequently described as age-related pathologies without any genetic linkage and with distinct clinical symptoms, they all share common degenerative mechanisms that converge on mitochondria. Most of these diseases exhibit metabolic defects and increased oxidative stress. For example, in Alzheimer’s disease (AD) there are significant changes in mitochondrial morphology and number (Hirai et al., 2001; Baloyannis, 2006), which are associated with reduced levels of some of the ETC subunits. Besides providing ATP, mitochondria sense localized Ca^2^^+^ changes and prevent the build-up of excessive intracellular Ca^2^^+^ that can trigger death programs. In a variety of neurodegenerative disorders, accumulation of glutamate at the synaptic cleft leads to prolonged neuronal depolarization and, through intracellular and plasma membrane Ca^2^^+^ permeable channels, large Ca^2^^+^ influx (Bano and Nicotera, 2007; Moskowitz et al., 2010). The sustained mitochondrial Ca^2^^+^ uptake leads to extensive mitochondrial depolarization and release of pro-death factors, which then promote caspase-dependent and independent cell death according to the intensity of the stimulus (Ankarcrona et al., 1995; Orrenius et al., 2003). Notably, at least *in vitro*, uncoupling of the mitochondrial ETC significantly reduces cell death as a result of the excitotoxic Ca^2^^+^ overload (Budd and Nicholls, 1996). Thus, at least for a limited period of time, mild mitochondrial dysfunction and time-limited collapse of the membrane potential can be protective against neurotoxins and favor neuronal survival.

## CAN LONGEVITY PATHWAYS CONFER NEUROPROTECTION?

Despite the large number of studies on aging in model organisms, especially invertebrates, there is still an open question: can pro-longevity pathways prevent brain disorders? Although more work is required to prove the relevance in humans, new evidence suggests that low insulin/IGF-1 signaling or decreased TOR signaling has a beneficial effect in aggregate-prone animal models of neurodegenerative diseases. More specifically, *Igf1r *haploinsufficiency can reduce inflammatory response, neuronal loss and cognitive impairment associated with toxic Aβ aggregates in mouse models of AD (Cohen and Dillin, 2008; Cohen et al., 2009; Freude et al., 2009; Killick et al., 2009). Over time, decreased IGF-1 levels promote the assembly of densely packed fibrils that are less toxic compared with Aβ oligomers. In line with this, activation of the DAF-16/FOXO3a, one of the main downstream targets of the insulin/IGF-1 signaling, either genetically – encoding a nuclear targeted FOXO3a – or pharmacologically – using a specific compound called Psammaplysene A (PA) – protects both *in vitro* and *in vivo* against insults causing motor neuron disease (Mojsilovic-Petrovic et al., 2009). Similarly to the insulin/IGF-1 signaling pathway, long-term rapamycin treatment prevents cognitive deficits throughout the lifespan in mice (Ehninger et al., 2009; Halloran et al., 2012). In an AD mouse model, rapamycin improves learning and memory, ameliorates cognitive defects, and slows or blocks the progression of the disease (Caccamo et al., 2010; Spilman et al., 2010). However, even in wild type mice, rapamycin seems to have a beneficial effect in cognition, since it can ameliorate learning and memory deficits (Majumder et al., 2012). In another interesting study, it was found that the oral administration of the natural polyphenol resveratrol in mice was enough to activate the metabolic sensor AMPK and reduce the cerebral Abeta levels and their deposition in the cortex (Vingtdeux et al., 2010).

Downregulation of the insulin/IGF-1 signaling pathway, in a *C. elegans* model for Huntington’s disease delays dramatically the polyQ toxicity and the protein aggregates and protects from neurodegeneration (Morley et al., 2002). In accordance with this, mice for Huntington’s disease harboring only one copy of the *IRS2* – the insulin receptor substrate that control the phosphorylation of the downstream PI3K – have improved motor performance and live longer compared with their littermates (Sadagurski et al., 2011). Importantly, some of the ameliorated phenotypes are the result of improved mitochondrial activity and decreased levels of oxidative stress. Clioquinol is a metal chelator that has been extensively used as a neuroprotective drug in Alzheimer’s, Parkinson’s, and Huntington’s models or even as a drug in patients, where it reduces the accumulation or the expression of the toxic proteins (Cherny et al., 2001; Kaur et al., 2003; Nguyen et al., 2005). This drug inhibits the activity of CLK-1, a mitochondrial protein, and mimics many of the phenotypes produced by reduction of its activity in nematodes and mice. This might indicate that clioquinol acts, at least partially, through the mitochondrial pathway that affects longevity (Wang et al., 2009).

Taken together, these findings demonstrate that genetic and pharmacological interventions that diminish the PI3K/Akt or TOR signaling cascade can attenuate some of the damaging effects associated with the expression of aggregate-prone peptides. As part of the mechanism, the maintenance of mitochondrial activity and resistance to oxidative stress can delay neuronal loss in animal models of human brain disorders. In principle, we can predict the delay of at least some aspects of neurodegenerative disorders by altering those signaling cascades that directly or indirectly control mitochondrial activity and therefore regulate the progression of aging in an organism. However, more studies are required to prove the relevance of these findings in human pathology (see **Table 1**).

**Table 1:** Molecular pathways affecting aging and neurodegeneration.

	Aging	Neurodegeneration	Can the longevity pathway confer Neuroprotection?
Insulin/IGF-1 signaling	+	?	YES
TOR pathway	+	?	YES
Mitochondria	+	+	?

*Insulin/IGF-1 and TOR signaling pathways are two of the main longevity determinants, whose downregulation not only increases the lifespan but also protects from neurodegenerative insults. Mitochondrial function is critical both for aging and neurodegeneration: mild dysfunction is beneficial and increases the lifespan of the organism, whereas a sustained deregulation leads to cell death.*

## CONCLUDING REMARKS

Over the last years, significant progress was achieved in the field of aging and led to the identification of molecular pathways underlying this important biological process. Most of these molecular pathways are evolutionarily conserved and affect various tissues, including the central nervous system. This is reflected by changes both in the morphology and the function of the neurons, which can promote cognitive decline and the onset of neurodegenerative diseases. Interestingly, some of the mechanisms that regulate aging are linked to neurodegeneration.

Mitochondrial activity significantly contributes to aging and plays a major role in neurodegeneration. However, these organelles influence in an opposite way these processes: severe mitochondrial dysfunction triggers neurodegeneration and affects animal survival (Gerlach et al., 1991; Kong and Xu, 1998; Panov et al., 2005; Keeney et al., 2006), whereas mild mitochondrial dysfunction prolongs the lifespan of various organisms through broad metabolic changes and the build-up of protective defenses against stressful conditions (Wong et al., 1995; Feng et al., 2001; Dillin et al., 2002; Lee et al., 2003; Liu et al., 2005; Dell’agnello et al., 2007; Copeland et al., 2009). Nevertheless, there are still many open questions that must be addressed. For example, what is the limit beyond which mitochondrial deficiency causes cell death? Is mitochondrial activity a good anti-aging target? Can modulation of mitochondrial function prolong life expectancy without causing neurodegeneration? The better understanding of the molecular mechanisms underlying aging might offer opportunities to improve healthy human lifespan and in parallel to provide new therapeutic strategies for brain disorders.

## Conflict of Interest Statement

The authors declare that the research was conducted in the absence of any commercial or financial relationships that could be construed as a potential conflict of interest.
